# Case Report: Extended Clinical Spectrum of the Neonatal Diabetes With Congenital Hypothyroidism Syndrome

**DOI:** 10.3389/fendo.2021.665336

**Published:** 2021-04-16

**Authors:** Vera Splittstoesser, Heike Vollbach, Michaela Plamper, Werner Garbe, Elisa De Franco, Jayne A. L. Houghton, Gesche Dueker, Rainer Ganschow, Bettina Gohlke, Felix Schreiner

**Affiliations:** ^1^ Pediatric Endocrinology Division, Children’s Hospital, University of Bonn, Bonn, Germany; ^2^ Department of Neonatology, St. Marien-Hospital, Bonn, Germany; ^3^ Institute of Biomedical and Clinical Science, University of Exeter Medical School, Exeter, United Kingdom; ^4^ Genomics Laboratory, Royal Devon & Exeter Hospital, Exeter, United Kingdom; ^5^ Division of Pediatric Gastroenterology and Hepatology, Children’s Hospital, University of Bonn, Bonn, Germany

**Keywords:** *GLIS3*, neonatal diabetes, anemia, exocrine pancreatic insufficiency, cholestasis, megalocornea

## Abstract

**Background:**

Neonatal diabetes with congenital hypothyroidism (NDH) syndrome is a rare condition caused by homozygous or compound heterozygous mutations in the GLI-similar 3 coding gene *GLIS3*. Almost 20 patients have been reported to date, with significant phenotypic variability.

**Case presentation:**

We describe a boy with a homozygous deletion (exons 5-9) in the *GLIS3* gene, who presents novel clinical aspects not reported previously. In addition to neonatal diabetes, congenital hypothyroidism and other known multi-organ manifestations such as cholestasis and renal cysts, he suffered from hyporegenerative anemia during the first four months of life and presents megalocornea in the absence of elevated intraocular pressure. Compensation of partial exocrine pancreatic insufficiency and deficiencies in antioxidative vitamins seemed to have exerted marked beneficial impact on several disease symptoms including cholestasis and TSH resistance, although a causal relation is difficult to prove. Considering reports on persistent fetal hemoglobin detected in a few children with *GLIS3* mutations, the transient anemia seen in our patient may represent a further symptom associated with either the *GLIS3* defect itself or, secondarily, micronutrient deficiency related to exocrine pancreatic deficiency or cholestasis.

**Conclusions:**

Our report expands the phenotypic spectrum of patients with *GLIS3* mutations and adds important information on the clinical course, highlighting the possible beneficial effects of pancreatic enzyme and antioxidative vitamin substitutions on characteristic NDH syndrome manifestations such as TSH resistance and cholestasis. We recommend to carefully screen infants with *GLIS3* mutations for subtle biochemical signs of partial exocrine pancreatic deficiency or to discuss exploratory administration of pancreatic enzymes and antioxidative vitamins, even in case of good weight gain and fecal elastase concentrations in the low-to-normal range.

## Background

Neonatal diabetes with congenital hypothyroidism (NDH) syndrome is a rare disorder caused by autosomal recessive mutations in the *GLI-similar 3 (GLIS3)* gene. Since the first description of two affected siblings in 2003 (1) and the identification of underlying *GLIS3* mutations in 2006 (2), almost 20 patients with NDH syndrome have been reported so far (3–5). GLIS3 is a Krüppel-like zinc-finger transcription factor expressed during early embryogenesis and considered to be important for the proper development of various organs (6, 7). In addition to the two cardinal symptoms neonatal diabetes and congenital hypothyroidism, reported patients frequently suffered from intrauterine growth retardation (IUGR), a typical facial phenotype, cholestasis, liver fibrosis, renal cysts and glaucoma. Further described symptoms include developmental delay, hearing disorder, exocrine pancreatic insufficiency, pancreatic and splenic cysts, osteopenia and cardiac anomalies, as well as single cases with choanal atresia, craniosynostosis and undervirilization of male external genitalia (3–5, 8). Phenotype distribution and severity of organ manifestations varies substantially between individual patients. Thyroid gland morphology, for example, varies from normal anatomy to athyroidism (4).

The first reported patients, two siblings from Saudi Arabia with a nonsense frameshift mutation (c.1873dupC), died in early infancy due to infections (1, 2). A third child from the same family, which was born later, died of the same condition (9). However, subsequent reports and follow up of known patients with *GLIS3* mutations revealed that even patients with a severe multiorgan phenotype and deletions spanning several exons of the *GLIS3* gene may have a longer life expectancy than originally described (4).

Here we report a patient with a homozygous deletion in the *GLIS3* gene who, in addition to neonatal diabetes, hypothyroidism and further previously reported organ manifestations, suffered from hyporegenerative anemia and megalocornea without elevated intraocular pressure. Furthermore, we discuss possible beneficial effects of pancreatic enzyme replacement and antioxidative vitamins A, C, E and K on characteristic NDH syndrome manifestations such as TSH resistance and liver disease.

## Case Report

**Figure 1:**
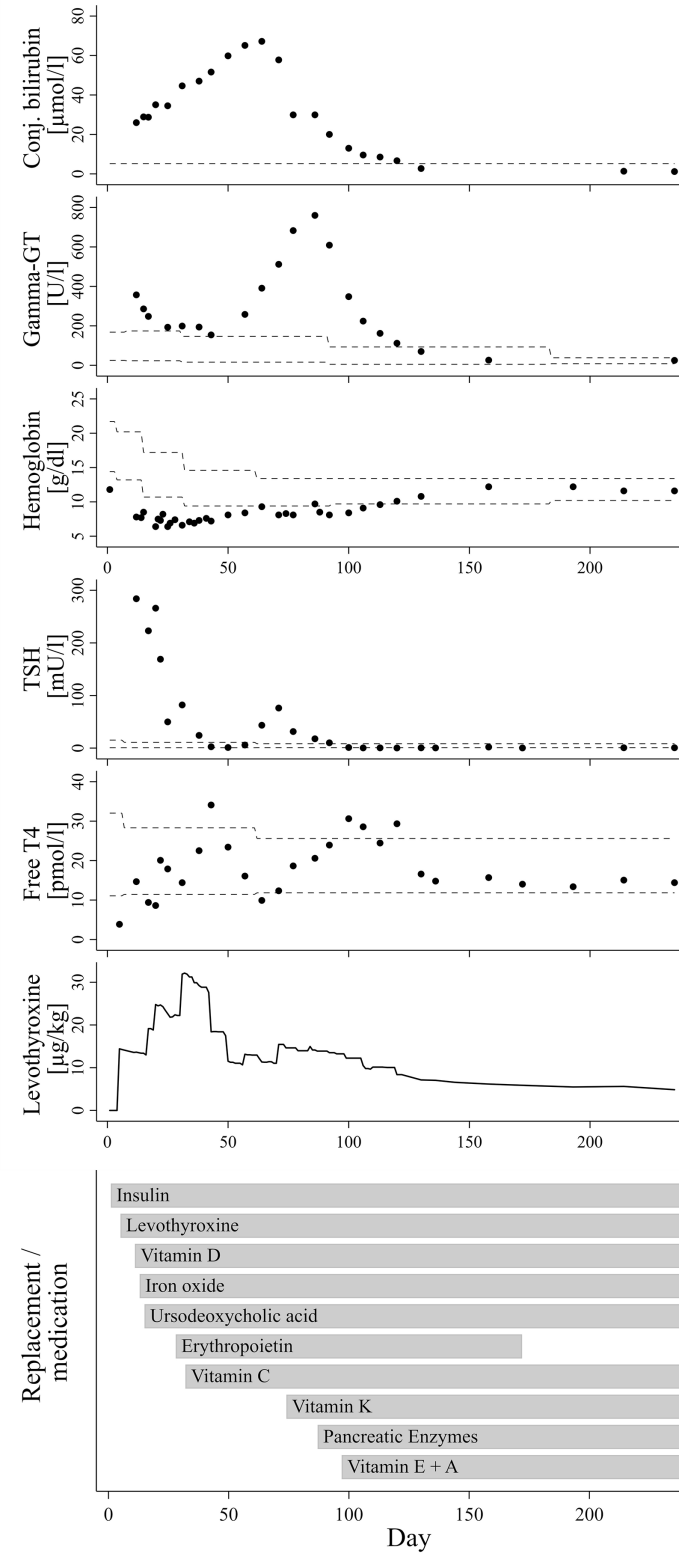
Course of laboratory parameters and treatment over time. The dashed lines indicate age-adjusted reference values.

The now seven months old boy was born in Germany after 37 weeks of gestation *via* cesarean section due to oligohydramnios and intrauterine growth retardation [birth weight 1995 g/-2.47 SDS (10)]. The parents are refugees from Syria, self-reported to be distantly related. On the first day of life, the boy presented hyperglycemia persistently >200 mg/dl. Insulin and C-peptide concentrations below detection limit (insulin <2 µU/ml; C-peptide <0.1 ng/ml) led to the diagnosis of neonatal diabetes mellitus and initiation of intravenous insulin substitution. Furthermore, newborn screening demonstrated a very high TSH (>250 µU/ml). Congenital hypothyroidism was confirmed by low fT4 levels (fT4 0.3 ng/dl), while ultrasound examination revealed normal thyroid gland architecture and volume. The coincidence of these endocrine entities, together with elevated conjugated bilirubin indicating cholestasis already in the first days of life and several small cysts detected by ultrasound in kidneys and pancreas led the suspicion of an underlying neonatal diabetes with congenital hypothyroidism (NDH) syndrome due to a *GLIS3* mutation. Normalization of TSH seemed difficult to achieve at this time, which was in accordance to previous reports on infants with NDH syndrome due to *GLIS3* mutations. Although serum levels for free thyroxine reached the lower normal range under substitution with approx. 15 µg/kg/d levothyroxine around day 11, TSH normalization was only reached at day 42 after successive increase of levothyroxine to 35 µg/kg/d, administered intravenously and divided into three doses daily (**Figure 1**).

In addition to neonatal diabetes, hypothyroidism, cholestasis and kidney cysts, the boy presented congenital hyporegenerative anemia (**Figure 1**; Hb at day one: 11.8 g/dl, Hb minimum around day 34: 6.5 g/dl), which has not been reported in individuals with homozygous *GLIS3* mutations to date. While under treatment with up to 4 mg/kg of iron oxide, hemoglobin levels further declined while reticulocyte count remained inappropriately low (max. 7.2 %/143.3 G/l during the first months of life). Serum concentrations of folic acid and vitamin B12 were normal. Serological tests for EBV, HHV6 and Parvovirus B19 as well as PCR for CMV in urine were negative. Because of stimulated erythropoietin serum concentrations not exceeding the upper normal range (day 21: 29.4 mU/ml; ref. 4.3-29 mU/ml), we hypothesized a limited erythropoietin synthesis due to impaired liver and/or kidney function and started substitution of recombinant erythropoietin (500 IE s.c. twice weekly). In addition, Vitamin C serum concentration also appeared to be diminished (1.1 to 2.2 mg/L; ref. 4.6-14.9 mg/L). However, combined substitution of iron, erythropoietin and Vitamin C only led to incomplete normalization of hemoglobin levels, while reticulocyte count remained inappropriately low for several weeks.

Under treatment with ursodeoxycholic acid (UDCA), which was started at day 15 of life, conjugated bilirubin levels further increased to a maximum of approx. 3.93 mg/dl at age nine weeks. Serum gamma glutamyl transferase activity presented a continuous rise from week six onwards to a maximum of 760 U/l at age 12 weeks. Alanine transaminase (ALT) and aspartate transaminase (AST) serum enzyme activities as well as concentration of albumin and cholinesterase activity were always within normal ranges. Similarly, growth in length and weight gain were appropriate for age, and stool did not look suspicious of fat malabsorption. Repeated analyses of fecal elastase revealed values just above the lower reference limit (104 and 150 µg/g at the age of two and six weeks, respectively), whereas serum lipase activities were normal (repeatedly at approx. 20 U/l). However, low serum concentrations of vitamins A, D and E as well as diminished coagulation indices for quick/INR and vitamin K-dependent factor activities then clearly indicated clinically relevant maldigestion, putatively as a consequence of both cholestasis and partial exocrine pancreatic insufficiency.

Initiation of vitamin K substitution at the age of ten weeks, followed by pancreatic enzyme replacement and substitution of further fat-soluble vitamins between weeks 10 to 14 of age not only stabilized blood coagulation factors, but also appeared to be related to a significant reduction of levothyroxine dose requirement and final resolution of cholestasis and anemia (**Figure 1**).

Ophthalmological examinations at age two weeks and four weeks revealed borderline enlarged corneae (diameter 11 mm), whereas intraocular pressure was normal. At age 12 weeks, a corneal diameter of 12 mm together with increased intraocular pressure (30 mmHg) directed suspicion to early onset glaucoma, as reported in several earlier patients with NDH syndrome. However, repeated examinations with optimized test conditions at age 13 and 19 weeks confirmed the finding of isolated megalocornea (diameter 12 mm) without elevated intraocular pressure (10-16 mmHg).

The boy is currently seven months old and in good health, showing a slight delay in gross motor development. Body length and weight are within upper normal range: length 72 cm [+0.97 SDS according to WHO growth charts (11)]; weight 9.8 kg (+1.37 SDS). Blood glucose concentrations mostly range between the age-adjusted target range of 100-200 mg/dl (~5.6–11.1 mmol/l) and modestly elevated levels under continuous subcutaneous insulin infusion *via* insulin pump device (total daily dose approx. 0.7 IU/kg/d; current HbA1c 8.7%). TSH and fT4 serum concentrations are normal under substitution with 4.9 µg/kg/d levothyroxine (50 µg once daily p.o.), whereas thyroglobulin levels remain elevated (263 ng/ml, ref. 1.7–55.6 ng/ml). Hearing test in the neonatal period was unremarkable. Facial gestalt included several typical aspects known from previously reported patients with *GLIS3* mutations (8): low-set ears, prominent eyes, long philtrum and thin vermillion border of the upper lip.

The patient has one healthy brother, five-years-old. Two further brothers died in Syria during the neonatal period. One of them died from unknown reason a few days after being born with a gestational age of eight months and a birth weight of approx. 2000 g. The other died after one week, with icterus and without having passed meconium (birth at term, approx. 2500 g).

## Genetic Analysis

Blood samples of the patient and its parents were analyzed by next generation panel sequencing for known neonatal diabetes loci at the Exeter Genomics laboratory, UK, as previously described (12). This assay can detect copy number variation using dosage analysis. The panel analysis included coding regions and conserved splice sites of *ABCC8, AGPAT2, BSCL2, CISD2, CNOT1, COQ2, COQ9, EIF2S3, EIF2AK3, FOXP3, GATA4, GATA6, GCK, GLIS3, HNF1B, IER3IP1, IL2RA, INS, INSR, KCNJ11, LPL, LRBA, MNX1, NEUROD1, NEUROG3, NKX2‐2, PDX1, PTF1A, RFX6, SLC2A2, SLC19A2, STAT3, WFS1* and *ZFP57* (https://www.diabetesgenes.org/about-neonatal-diabetes/genetic-testing-for-neonatal-diabetes/). A homozygous *GLIS3*-deletion of exons 5 to 9 was identified in our patient (NM_001042413.1:(c.1710+1_)?_(?_c.2474-1),p.?, deletion of exons 5 to 9). Both parents are heterozygous carriers of this deletion.

## Discussion

We describe a boy with a homozygous deletion in the *GLIS3* gene, who presents novel clinical aspects not reported previously in almost 20 patients with NDH syndrome. In addition to the two cardinal symptoms neonatal diabetes and congenital hypothyroidism and further known findings such as IUGR, cholestasis and renal cysts, our patient suffered from hyporegenerative anemia during the first four months of life and has megalocornea in the absence of elevated intraocular pressure. Furthermore, compensation of a partial exocrine pancreatic insufficiency and deficiencies in antioxidative vitamins seemed to have exerted marked beneficial impact on several disease symptoms including cholestasis and TSH resistance, although a causal relation is difficult to prove.

Around the age of nine weeks, both total and conjugated bilirubin serum concentration reached a peak and started to decrease. At this time, course of fT4 serum levels showed a nadir (see **Figure 1**), after which the daily levothyroxine dose was readjusted. However, fT4 serum levels returned to the medium to upper normal range only two to three weeks thereafter, meaning that normalization of thyroid hormone levels is unlikely to represent a dominant factor triggering this trend change towards resolution of cholestasis. Initiation of vitamin K substitution seemed to be more closely related, because a first significant decrease of serum bilirubin levels was noted only a few days thereafter (**Figure 1**). This may indicate that compensation of vitamin K deficiency, which is a common finding in cholestatic patients (13), could have exerted beneficial influence on liver function and/or bilirubin excretion itself. Indeed, there is experimental evidence for the assumption of liver protective vitamin K effects in conditions of extrahepatic cholestasis, presumably *via* attenuation of pathological activation and transformation of hepatic stellate cells (14). However, gamma glutamyl transferase (GGT) serum activity in our patient continued to rise from 391 U/l at week nine of age to its peak at 760 U/l about three weeks later, hereby clearly exceeding the expected delay due to differences in half-life between bilirubin and GGT serum activity. Notably, a few days after initiation of pancreatic enzyme substitution around week 12, GGT activity started to sharply decrease and then normalized over the following four weeks, as did bilirubin levels (**Figure 1**). We therefore speculate that partial exocrine pancreatic enzyme deficiency, even if measurements of fecal elastase were only slightly below age-adjusted normal ranges in our patient, could be a relevant cofactor in the etiology of cholestatic liver disease, probably in concert with low concentrations of antioxidant and cytoprotective vitamins.

Out of 19 patients with NDH syndrome due to *GLIS3* mutations reported to date, exocrine pancreatic insufficiency is documented in four cases, all of which suffer from hepatic disease with liver fibrosis (3, 4). Six of 19 patients have been described to have no impairment of liver function or architecture, and none of them has known exocrine pancreatic insufficiency (2, 4). Consequently, there are nine remaining cases with NDH and liver diseases, but without known exocrine pancreatic insufficiency. We suggest it might be worth not only to carefully screen for subtle biochemical signs of partial exocrine pancreatic deficiency but also to discuss exploratory administration of pancreatic enzymes and/or antioxidative vitamins in those patients. On the other hand, the four reported patients with known exocrine pancreatic insufficiency and enzyme substitution are all reported to suffer from significant chronic liver pathologies such as fibrosis or hepatitis. Consequently, liver function and bile flow parameter have to be regularly monitored in our patient in order to detect relapse of cholestatic liver disease.

Besides vitamin K (in step-by-step reduction, current vit. K1 serum concentration 7850 ng/l; ref. 50-1800 ng/l) our patient is still being administered antioxidative vitamins A, C and E. Current serum concentrations for vitamins A (27.7 µg/dl), C (13.5 mg/l) and E (1.62 mg/dl) are in the mid to upper tertile of the normal range [for age-adjusted ref. ranges for vitamins A and E see (15)]. Interestingly, *Gulo*(-/-) mice, which are unable to synthesize vitamin C, were more susceptible to liver damage and hepatic fibrosis induced experimentally by lithocholic acid feeding as compared to wildtype animals, while in *Gulo*(-/-) mice supplemented with vitamin C hepatic fibrosis was significantly attenuated (16). Hepatoprotective effects have been demonstrated under experimental conditions also for vitamins A, E and K (14, 17, 18). However, meta-analyses of randomized clinical trials using antioxidant vitamins among patients with various chronic liver disorders did not reveal consistent evidence of therapeutical hepatoprotective effects (19), with modest beneficial effects only for vitamin E (20, 21).

Persistently elevated levels of TSH despite normalization of fT4 and use of high levothyroxine doses up to 75 µg/kg/day have been reported in several earlier NDH syndrome case descriptions (2–5). The reason for this TSH resistance is currently unclear. There is also no clear correlation between the degree of TSH resistance and morphological abnormalities of the thyroid gland or even athyreosis, which is observed in some NDH patients, including an Arab girl with a similar deletion of *GLIS3* exons 5-9 (4) as in the present case. Substitution dose in our patient was gradually increased to up to 35 µg/kg/d in the fourth week of life, in order to normalize both fT4 and TSH. Normal serum concentrations for both total T3 and fT3, which were repeatedly determined between the second and fourth week of life, ruled out underlying deiodinase insufficiency. Later on, each step of the successive replacement of vitamins (especially vitamin C and K) and pancreatic enzymes seemed to support final normalization of TSH levels with usual age-appropriate substitution doses of approx. 5 µg/kg/d levothyroxine (**Figure 1**). Indeed, there is some evidence to suggest that vitamin C may enhance the intestinal absorption of levothyroxine (22, 23). Furthermore, in a case series of ten infants with newly diagnosed cystic fibrosis and hyperthyrotropinemia, pancreas enzyme replacement normalized elevated TSH levels without levothyroxine substitution in nine of ten patients (24). The authors discuss a correlation with improvement of the nutritional status, including a better resorption of iodine and selenium. Iodine concentrations were not analyzed in our patient. However, a normal selenium concentration of 73.6 µg/l (Ref. 53-105 µg/l), which was retrospectively determined in a serum sample taken only a few days after initiation of pancreatic enzyme replacement, rules out a causal role of selenium levels. Interestingly, Hiss and Dowling demonstrated in patients with exocrine pancreatic insufficiency that fecal losses of thyroxine were considerably greater than normal, even under enzyme replacement therapy, and speculated on an impairment of the enterohepatic circulation of thyroxine. Because net peripheral tissue turnover of thyroxine was within normal range, they concluded that a compensatory increase in hormone synthesis by the thyroid gland must have occurred in response to the higher fecal losses (25). Furthermore, bile itself has been shown to facilitate thyroxine absorption from intestinal lumen in rats, probably due to a bile component that reduces binding capacity of thyroxine with intraluminal plasma proteins (26). These effects might in part explain the unusually high levothyroxine requirement and the TSH resistance seen in some patients with *GLIS3* mutations, which are prone to exocrine pancreatic insufficiency and cholestasis. On the other hand, persistently elevated thyroglobulin serum levels even in euthyroid conditions with low-to-normal TSH concentrations, as seen in our patient, indicate the presence of different pathophysiological mechanisms during earlier stages of thyroid hormone synthesis and metabolism.

Our case report is the first describing a congenital hyporegenerative anemia in a patient with a *GLIS3* mutation. Initially, we hypothesized a limited erythropoietin synthesis due to impaired liver and/or kidney function. However, hemoglobin and reticulocyte count remained low for several months, even under combined substitution of erythropoietin, iron and vitamin C. Considering the widespread expression of the transcription factor GLIS3 among different organs and progenitor cell populations (27) as well as findings on persistent fetal hemoglobin levels detected in two earlier reported children with *GLIS3* mutations (4, 5), it is possible that the transient hyporegenerative anemia may represent a further symptom related to the *GLIS3* defect. On the other hand, at the age of five months, when hemoglobin concentration had normalized, hemoglobin electrophoresis did not reveal age-inappropriately high levels of fetal hemoglobin (HbF 3.2 %; HbA0 >80 %) in our patient. As well as the majority of patients with homozygous *GLIS3* mutations, *GLIS3*-deficient mice (*GLIS3* ^-/-^), who present a similar phenotype with impaired pancreatic islet development, polycystic kidney disease and hypothyroidism, did not exhibit overt congenital hematological problems (28). Thus, the transient congenital anemia seen in our patient might also be a coincidental finding related to another genetic variant, especially in consideration of the parental consanguinity. Whole exome sequencing, which was initially discussed in view of the complex phenotype including severe hyporegenerative anemia, was not pursued after confirmation of the homozygous deletion in the *GLIS3* gene and later on normalization of hemoglobin levels.

As opposed to approximately 50 percent of the so far reported patients with NDH syndrome who suffer from congenital or early onset glaucoma (1, 2, 4, 5), our patient is, to our knowledge, the first with isolated megalocornea in the absence of elevated intraocular pressure. Theoretically, the reported number of infants with congenital glaucoma may be overestimated due to difficult examination conditions especially in neonates and young infants. Second, comparatively high serum concentrations for antioxidative vitamins in our patient could represent a protective factor, although evidence for a beneficial impact on intraocular pressure and (open angle) glaucoma risk is limited and may only involve vitamins A and C (29). However, a widely distributed *GLIS3* expression detected in several ocular compartments and recent GWAS findings linking a *GLIS3* polymorphism to primary angle closure glaucoma (30) clearly indicate that regular ophthalmological monitoring is necessary also in those patients without early diagnosed glaucoma.

## Conclusion

Our report expands the phenotypic spectrum of patients with *GLIS3* mutations and adds important information on the clinical course, with possible beneficial effects of pancreatic enzyme and antioxidative vitamin substitutions on characteristic NDH syndrome manifestations such as TSH resistance and cholestasis. Even in case of good weight gain and fecal elastase concentrations in the low-to-normal range, we recommend to carefully screen for subtle biochemical signs of partial exocrine pancreatic deficiency or to discuss exploratory administration of pancreatic enzymes and antioxidative vitamins.

## Data Availability Statement

The raw data supporting the conclusions of this article will be made available by the authors, without undue reservation.

## Ethics Statement

Ethical review and approval was not required for the study on human participants in accordance with the local legislation and institutional requirements. Written informed consent to participate in this study was provided by the participants’ legal guardian/next of kin.

## Author Contributions

VS, HV, MP, WG, GD, RG, BG and FS participated in the diagnostic workup and interpretation of clinical and biochemical data. EDF and JH performed and interpreted the genetic analysis. VS and FS wrote the manuscript. All authors contributed to the article and approved the submitted version.

## Funding

Genetic testing was funded by a Wellcome Trust Senior Investigator Award to Professors Andrew Hattersley and Sian Ellard, Exeter, UK. EDF is a Diabetes UK RD Lawrence fellow in Diabetes Research.

## Conflict of Interest

The authors declare that the research was conducted in the absence of any commercial or financial relationships that could be construed as a potential conflict of interest.

## Abbreviations

NDH, neonatal diabetes with congenital hypothyroidism; *GLIS3*, *GLI-similar* 3; GGT, gamma glutamyl transferase; GWAS, genome wide association study.
